# Clinical and Pathological Analysis of 4910 Patients Who Received Renal Biopsies at a Single Center in Northeast China

**DOI:** 10.1155/2019/6869179

**Published:** 2019-03-26

**Authors:** Ping Nie, Rui Chen, Manyu Luo, Changqing Dong, Liangmei Chen, Juan Liu, Liangqian Hu, Bing Li, Ping Luo

**Affiliations:** ^1^Department of Nephropathy, The Second Hospital of Jilin University, Changchun, Jilin Province 130041, China; ^2^School of Preclinical Medicine, Changchun University of Chinese Medicine, Changchun, Jilin Province 130117, China

## Abstract

**Purpose:**

To identify the epidemiology and pathological types of kidney diseases and their changes during the past decade, in a population from Northeast China.

**Methods:**

We retrospectively analysed clinical and renal pathological data from 4910 patients who received renal biopsies in the Second Hospital of Jilin University from 2008 to 2017.

**Results:**

Males received more renal biopsies than females (*p* < 0.001). The average age (*p* < 0.001) and percentage of elderly patients (*p *< 0.001) increased over time. The pathological types were primary glomerulonephritis (PGN, 73.2%), secondary glomerulonephritis (SGN, 23.7%), tubular-interstitial nephropathy (TIN, 2.8%), and hereditary nephropathy (HN, 0.3%). The most common forms of PGN were membranous nephropathy (MN, 37.2%) and IgA nephropathy (IgAN, 29.9%). Over time, the prevalence of IgAN decreased, but the prevalence of MN increased. MN was more common in middle-aged and elderly patients, but IgAN was most common in young adults. Analysis of SGN data indicated that lupus nephritis (LN, 34.0%), Henoch-Schönlein purpura glomerulonephritis (HSPN, 17.9%), and diabetic nephropathy (DN, 11.7%) were the most common forms. Over time, the prevalence of DN (*p* = 0.003), hypertension-associated renal damage (*p* = 0.005), and systemic vasculitis-associated nephritis (SVARD,* p *< 0.001) increased, but the prevalence of HSPN (*p* < 0.001) and hepatitis B virus-associated glomerulonephritis (HBV-GN,* p *= 0.001) decreased. Nephrotic syndrome was the main clinical manifestation of PGN.

**Conclusion:**

From 2008 to 2017, renal biopsies were increasingly performed in the elderly. There were notable changes in the epidemiology and pathological types of kidney disease among renal biopsy patients at our centre.

## 1. Introduction

The prevalence of chronic kidney disease (CKD) has continued to increase in recent years, and it currently affects 10 to 16% of the general adult populations in Asia, the United States, Australia, and Europe [1]. A cross-sectional survey of CKD among adults in China during 2012 showed that the prevalence was 10.8% [2]. Early intervention is important for these patients because it can delay disease progression and reduce the incidence and mortality of end-stage renal disease (ERSD). Iversen and Brun performed the first percutaneous renal biopsy in 1951 [3]. This technique is now the gold standard for diagnosis of renal disease.

The prevalence of different pathological types of renal diseases varies with patient age, geographic origin, and ethnic group. Recent studies showed that primary glomerulonephritis (PGN) is the most common glomerulonephritis in China in that it accounts for 61.7 to 74.0% of all incident cases [4–9]. In recent years, the incidence of secondary glomerulonephritis (SGN) has increased in China [7]. Many reports in China indicated that IgA nephropathy (IgAN) is the most common type of PGN [4–10], and studies in Japan, Europe, and Oceania had similar findings [11–15]. Studies in Africa [16] and India [17] indicated that minimal change disease (MCD) and focal segmental glomerulosclerosis (FSGS) were the most common nephropathies, but studies in the United States [18, 19] and South America [20] indicated that FSGS was the most common nephropathy. There are also reports of recent increases in the incidence of membranous nephropathy (MN) [4–7, 9, 21]. The aging of the world population, the increasing burdens of environmental pollution, and the widespread use of pesticides and food additives may account for the increasing prevalence of kidney disease overall and for changes in the spectrum of kidney diseases [22, 23].

There is limited information of the pathological types of renal diseases in Northeast China from large samples of renal biopsy data. We analysed the age, sex, and pathological types of renal diseases and the relationship between pathological type and clinical diagnosis of 4910 patients who received renal biopsies in our centre from January 1, 2008, to December 31, 2017. We also compared the pathological types of renal disease from 2008 to 2012 (period 1) with the types from 2013 to 2017 (period 2).

## 2. Patients and Methods

Data of 4910 patients who received renal biopsies at the Second Hospital of Jilin University from 2008 to 2017 were retrospectively analysed. All patients were from Northeast China, and most were from Jilin Province. All enrolled patients received colour Doppler ultrasound guided percutaneous renal biopsy for collection of pathological specimens. Each patient signed an informed consent agreement before the biopsy that allowed use of their clinical and pathological data for scientific study. Institutional Review Board approval (2018198) was provided prior to study onset.

The biopsy specimens were processed and stained with haematoxylin and eosin (H&E), periodic acid-Schiff (PAS), periodic acid-Schiff-methenamine (PASM), and Masson's trichrome solution and then examined under an optical microscope (Olympus, U-MDOB3, Tokyo, Japan). Specimens from patients more than 40 years-old were also routinely stained with Congo red.

For immunofluorescence analysis, frozen renal tissues were stained for immunoglobulins (IgA, IgM, and IgG), complement components (C3, C4, and C1q), and fibrinogen. When necessary, staining was also performed for hepatitis antigens (HBsAg, HBcAg, and HBeAg) and for *κ* and *λ* light chains. Specimens collected before November 2014 were sent to the Electron Microscope Room of the Peking University First Hospital for analysis. After that time, all specimens were examined by a JEM-1400 Plus electron microscope in our department.

All diagnoses were according to the 1995 WHO histological classification of glomerular diseases. Thus, PGN included IgAN, mesangial proliferative glomerulonephritis (MsPGN), MN, focal segmental glomerulosclerosis (FSGS), minimal change disease (MCD), glomerular minor lesion (GML), crescentic glomerulonephritis (CreGN), membranoproliferative glomerulonephritis (MPGN), endocapillary proliferative glomerulonephritis (EnPGN), proliferative sclerosis, and sclerosing glomerulonephritis. SGN included HSPN, LN, DN, amyloidosis nephropathy (AN), light chain deposition nephropathy (LCDD), C3 glomerulopathy (C3GN), obesity-associated glomerulopathy (OAG), hypertension/malignant hypertension-associated renal damage (HT/MHTARD), SVARD, HBV-GN, hepatitis C virus associated nephritis (HCV-GN), and other minor conditions. TIN included acute interstitial nephritis (AIN), acute tubular necrosis (ATN), chronic interstitial nephritis (CIN), and subacute tubulointerstitial lesions. HN included thin basement membrane nephropathy (TBMN), Alport syndrome, Fabry disease, and collagen III glomerulopathy.

The clinical manifestations of PGN were acute glomerulonephritis syndrome (AGN), chronic glomerulonephritis syndrome (CGN), rapidly progressive glomerulonephritis syndrome (RPGN), nephrotic syndrome (NS), and asymptomatic urinary abnormality (AUA). The clinical manifestations of AGN were haematuria, red blood cell casts, proteinuria (<3.5 g/day), and high blood pressure for less than 3 months. The clinical manifestations of CGN were haematuria and proteinuria (1 to 3.5 g/day) for 3 or more months. The clinical manifestations of RPGN were acute nephritis syndrome with rapid progression of renal dysfunction. NS was defined as proteinuria of 3.5 g/day or more and plasma albumin level below 30 g/L. AUA was defined as haematuria and proteinuria (<1.0 g/day), identified during routine check-up and without edema or hypertension.

To analyse changes of renal disease diagnoses over time, patients diagnosed from 2008 to 2012 (period 1) were compared with those diagnosed from 2013 to 2017 (period 2). To analyse the effects of age on pathological types of kidney disease, patients were divided into 4 age groups (≤24 years, 25 to 44 years, 45 to 59 years, and ≥60 years).

SPSS version 21.0 (Chicago, IL, USA) was used for data analysis. Patient ages are presented as means ± standard errors and compared using the* t*-test. Categorical variables are presented as percentages and compared using the chi-square test. Differences in the pathological types of renal diseases between males and females and between periods 1 and 2 were analysed using a chi-square test. A* P*-value below 0.05 was considered significant.

## 3. Results

### 3.1. Pathological Types of Kidney Disease

We examined 4910 renal biopsy specimens from 2008 to 2017 (Table 1). There were 3593 cases of PGN (73.2%), 1165 cases of SGN (23.7%), 139 cases of TIN (2.8%), and 13 cases of HN (0.3%). There was no significant difference in the prevalence of these pathological types of renal diseases between period 1 and period 2 (*χ*^2^ = 4.566,* p* = 0.206).

**Figure 1:**
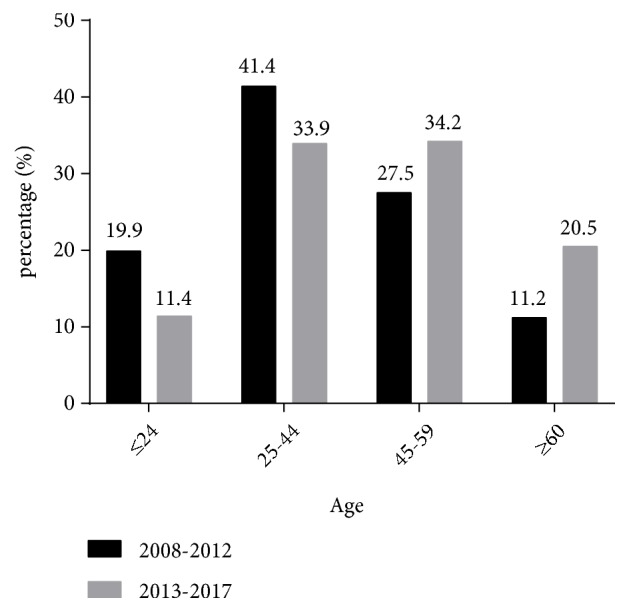
Percentages of patients diagnosed with kidney diseases who were in different age groups during different periods (n = 4910).

**Figure 2:**
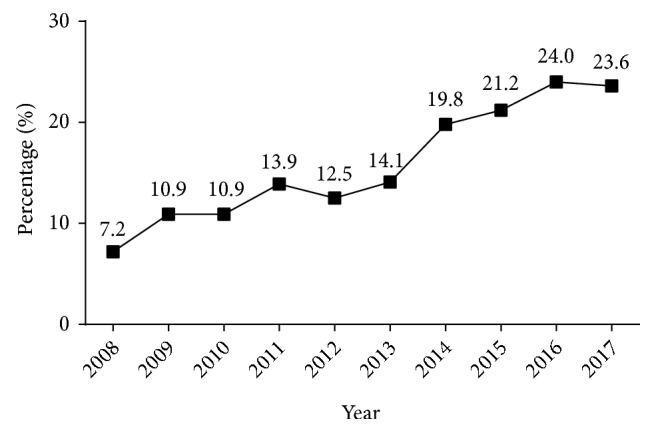
Percentage of patients diagnosed with kidney disease who were elderly (≥60 years-old), from 2008 to 2017.

There were 2629 males and 2281 females (ratio: 1.15:1) and significant differences in the gender ratios (*χ*^2^ =56.417,* p <* 0.001) for the different pathological types of renal disease (1:0.78 for PNG, 1:1.26 for SGN, 1:0.60 for TIN, and 1:1.60 for HN). The mean age was 42.6 ± 15.7 years (range: 7 to 84 years) and average age increased significantly over time (period 1: 39.8 ± 15.4 years, period 2: 45.4 ± 15.6 years,* p* < 0.001). Analysis of the four different age groups indicated that 37.6% of patients were 25 to 44 years old, 30.9% were 45 to 59 years-old, 15.9% were 60 years-old or more, and 15.6% were 24 years-old or younger (Figure 1). In addition, the proportion of patients who were 60 years-old or more increased significantly over time, from 7.2% in 2008 to 23.6% in 2017 (Figure 2).

### 3.2. Changes over Time in Pathological Types of Kidney Diseases

**Figure 3:**
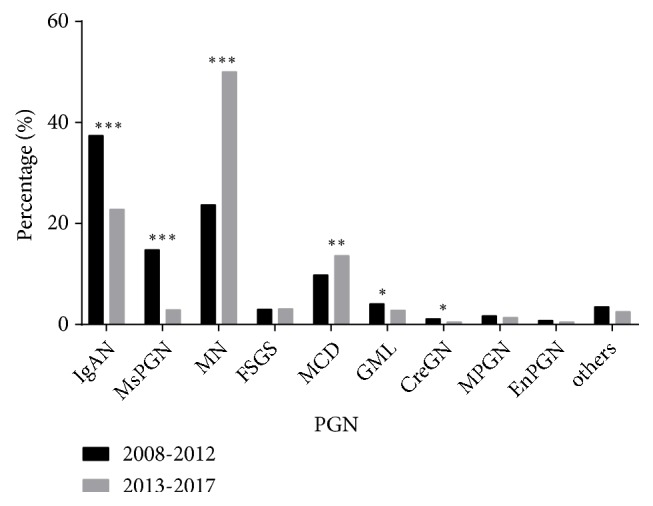
Pathological types of PGN during different periods. ^*∗*^*p* < 0.05, ^*∗∗*^*p* < 0.01, and ^*∗∗∗*^*p* < 0.001 by the chi-square test. Abbreviations:* IgAN*: IgA nephropathy;* MN*: membranous nephropathy;* MsPGN*: mesangial proliferative glomerulonephritis;* MCD*: minimal change disease;* GML*: glomerular minor lesion;* FSGS*: focal segmental glomerulosclerosis;* MPGN*: membranoproliferative glomerulonephritis;* EnPGN*: endocapillary proliferative glomerulonephritis;* CreGN*: crescentic glomerulonephritis; others: proliferative sclerosis and sclerosing glomerulonephritis.

**Figure 4:**
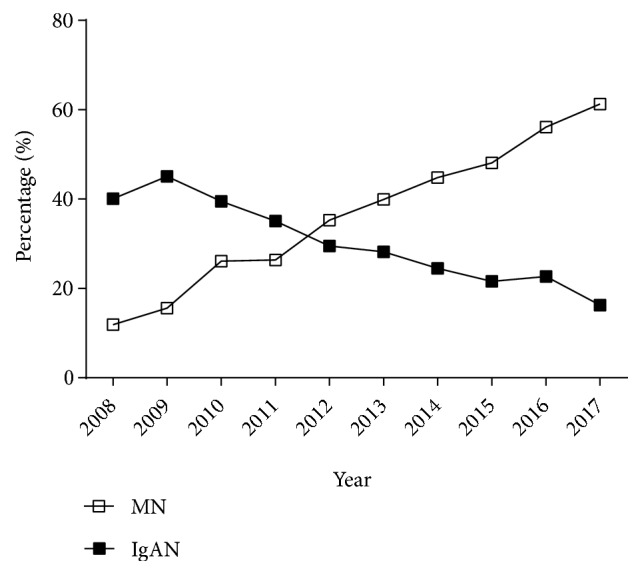
Changes in the percentages of PGN cases with MN and IgAN, from 2008 to 2017.

The most common types of PGN were MN (37.2%), IgAN (29.9%), MCD (11.7%), MsPGN (8.7%), GML (3.4%), and FSGS (3.1%) (Figure 3). In addition, from period 1 to period 2 there were significant decreases in the prevalence of IgAN (*p* < 0.001), MsPGN (*p *< 0.001), GML (*p* = 0.026), and CreGN (*p* = 0.047) but significant increases in the prevalence of MN (*p* < 0.001) and MCD* (p *= 0.001). Interestingly, the percentage of IgAN cases declined over time, from 45.1% in 2009 to 16.3% in 2017, but the percentage of MN cases increased over time, from 11.9% in 2008 to 61.3% in 2013 (Figure 4).

**Figure 5:**
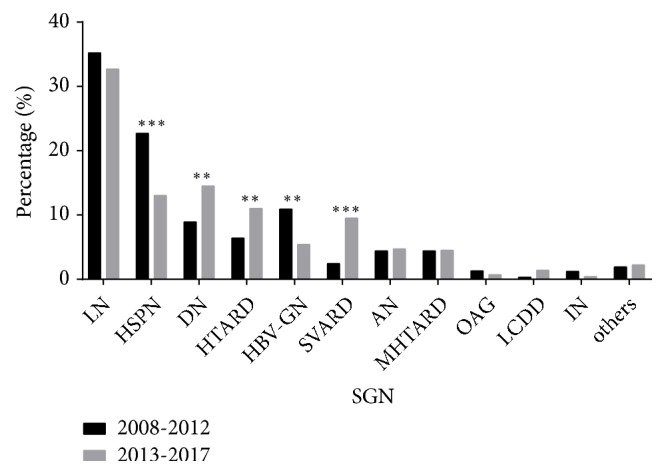
Pathological types of SGN during different periods. ^*∗*^*p* < 0.05, ^*∗∗*^*p* < 0.01, and ^*∗∗∗*^*p* < 0.001 by the chi-square test.* Abbreviations. LN*: lupus nephritis; HSPN: Henoch-Schönlein purpura nephritis;* DN*: diabetic nephropathy;* HT/MHTARD*: hypertension/malignant hypertension-associated renal damage;* HBV-GN*: hepatitis B virus associated nephritis;* SVARD*: systemic vasculitis-associated renal damage;* AN*: amyloidosis nephropathy;* OAG*: obesity-associated glomerulopathy;* LCDD*: light chain deposition nephropathy;* IN*: ischemic nephropathy; others:* SSARD*: Sjogren's syndrome associated renal damage;* MCTARD:* mixed connective tissue disease associated renal damage;* HUS*: hemolysis aemolytic uraemic syndrome;* C3GN*: C3 glomerulopathy;* LPN*: lipoprotein glomerulopathy;* CUAN*: chronic uric acid nephropathy;* ING*: idiopathic nodular glomerulosclerosis.

The most common SGN was systemic disease induced renal injury (52.7%), followed by vascular disease (19.8%), metabolic disease (19.1%), and infectious disease (8.4%) (Table 2, Figure 5). Moreover, the prevalence of each of these changed significantly from period 1 to period 2 (*p* < 0.001,* p* < 0.001,* p* = 0.004, and* p* = 0.001). Analysis of SGN cases indicated the main types induced by systemic diseases were LN (34.0%) and HSPN (17.9%); the main types induced by vascular diseases were HTARD (8.7%), SVARD (5.8%), and MHTARD (4.4%); the main type induced by metabolic disease was DN (11.7%); and the main type induced by infectious disease was HBV-GN (8.2%). Moreover, from period 1 to period 2 there were significant increases in the percentages of DN (*p* = 0.003), HTARD (*p* = 0.005), and SVARD (*p* < 0.001) but significant decreases in the percentages of HSPN (*p* < 0.001) and HBV-GN (*p* = 0.001). The prevalence of LN, AN, and other types had only small changes over time.

There were 139 cases of TIN (2.8%; Table 1). Further analysis (data not shown) indicated the most common TIN was subacute tubulointerstitial lesion (36.0%), followed by ATN (31.7%), AIN (23.7%), and CIN (7.9%). There was only one case of IgG4-related kidney disease. There were 13 cases of HN (0.3%; Table 1), and this included 7 cases of TBMN, 5 cases of Alport syndrome, and 1 case of collagen III glomerulopathy. Because of the small numbers of these cases, we did not analyse their changes over time.

### 3.3. Pathological Types of Kidney Disease in Different Age Groups

Analysis of the pathological types of kidney diseases in the different age groups indicated that MN was the most common kidney disease among patients aged 45 years and older (Table 3). The prevalence increased over time in three of the four age groups (25 to 44 years, 45 to 59 years, and 60 or more years,* p* < 0.001 for each comparison). IgAN had the highest prevalence in patients younger than 45 years, and its prevalence decreased significantly over time for two of the age groups (25 to 44 years and 45 to 59 years,* p* < 0.001 for each comparison). Comparison of periods 1 and 2 for patients aged 45 to 59 years indicated that the prevalence of MN had nearly doubled but the prevalence of IgAN was about half (*p* < 0.001 for each comparison). Except for IgAN, MCD was the most common kidney disease in patients aged 24 years and younger, and the prevalence nearly doubled from period 1 to period 2 (*p* < 0.001). MCD was also common in patients older than 60 years. For each age group, the prevalence of MsPGN declined over time. LN was the most common SGN in patients younger than 45 years. HSPN mostly occurred in patients younger than 25 years. The prevalence of HSPN decreased significantly over time for patients who were 25 to 44 years-old (*p* = 0.002) and 45 to 59 years-old (*p* = 0.028). Hypertensive renal damage occurred mostly in patients older than 25 years. DN was more common in patients older than 25 years, and the prevalence nearly doubled from period 1 to period 2 for those between 25 and 44 years-old.

### 3.4. Clinical Syndromes of PGN

The major clinical manifestations of PGN were NS (55.1%), followed by CGN (40.0%), AUA (3.4%), RPGN (0.8%), and AGN (0.7%) (Table 4). The main pathological types of NS were MN (51.8%), MCD (21.3%), MsPGN (10.8%), and IgAN (9.9%). IgAN (55.6%) and MN (21.2%) were the main pathological types of CGN. All RPGN cases were from CreGN, and the main cause of AGN was EnPGN (88.5%). IgAN (63.1%) and GML (25.4%) were the most common pathological types of AUA. SGN was mainly from LN (34.0%), HSPN (17.9%), DN (11.7%), and HBV-DN (8.2%). Because the etiologies of SGN, TIN, and HN are clear and these pathological types correspond to the clinical diagnoses, we did not analyse these data.

## 4. Discussion

We analysed the clinical and pathological data of 4910 patients who received renal biopsies from 2008 to 2017 at a single centre in Northeast China. Similar to many previous studies, most of our patients were male (53.5%) [4, 6, 7, 24]. In addition, most of our patients were 25 to 59 years-old (68.5%), indicating a predominance of young and middle-aged patients. We also found that the average age of renal biopsy patients increased during the past decade. In particular, the percentage of elderly patients (≥60 years-old) increased from 7.2% in 2008 to 23.6% in 2017. Other studies reported similar increases of age in patients receiving renal biopsies [6–8]. This is probably because of the increased use of this well-established procedure, the declining incidence of complications, and improvements in overall medical care for the elderly in China.

A recent systematic review identified 23 studies that examined more than 170,000 patients in China and reported that the prevalence of PGN and SGN were 74.0% and 22.1%, respectively [9]; this is consistent with our results (PGN: 73.2%, SGN: 23.7%). In our study, the percentage of MN increased over time and it became the most common pathological type of PGN, followed by IgAN, MCD, and MsPGN. We also found that the prevalence of IgAN, MsPGN, CreGN, and GML declined over time, but the prevalence of MN and MCD increased, consistent with most reports in China [9]. In our study, IgAN was the most common type of PGN between 2008 and 2012, and it accounted for 45.09% of cases during 2009, consistent with previous reports [4–10]. Similar to several recent reports, our prevalence of IgAN declined over time [9, 25]. Our analysis of different age groups indicated that IgAN had the highest prevalence among individuals younger than 45 years, in agreement with an American report which showed that IgAN was as common as FSGS in young adults (20 to 39 years-old) [18]. We also found that the rate of IgAN decreased significantly over time for individuals who were 25 to 59 years old (especially for those who were 45 to 59 years old), and its prevalence during period 2 was nearly half of that during period 1.

Some studies showed that the most common pathological type of kidney disease in the elderly was MN [26–29] and that the incidence of MN has increased over time [4–7, 9, 21]. In contrast, the prevalence of MN decreased over time in the United Kingdom [12] and Japan [30]. In our study, MN was the most common kidney disease since 2012 and was the most common PGN among patients older than 45 years. Its prevalence increased over time for those who were 25 to 44, 45 to 59, and 60 or more years-old. The increasing prevalence of MN in China may be due to the increasing number of elderly patients receiving renal biopsies, the increasing effects of environmental pollution, or the overuse of chemical agents during the recent acceleration of industrialization. One study in China reported that long-term exposure to high levels of fine particulate matter (PM2.5) increased the risk of MN [31]. In addition, the increasing use of immunofluorescence assays and electron microscopy has improved the diagnosis of early MN.

MCD is another common type of PGN. We found an increase in the detection rate of MCD over time, especially in younger people. Some renal biopsy specimens that had only minor lesions were not examined by electron microscopy before 2014. MCD is a podocytopathy with clinical manifestations of NS or massive proteinuria. Therefore, we diagnosed patients who had massive proteinuria as having MCD and those with less proteinuria as having GML. The prevalence of MsPGN declined over time. This is mainly because of progress in nephrology and pathological analyses during the past 10 years and the current classification of MsPGN into several types (mesangial proliferative IgAN, mesangial proliferative LN, etc.) [4, 8, 9].

LN was the most common pathological type of SGN in our population, generally consistent with many previous reports [5, 9, 13, 16, 17, 24]. Over time, the prevalences of DN, HTARD, and SVARD increased, and the prevalence of HSPN and HBV-GN decreased. Abundant evidence indicates that the prevalence of diabetes has increased significantly in most populations worldwide [9, 20, 32]. In addition, CKD related to diabetes has become more common than CKD related to glomerulonephritis in China [33]. Patients with a high clinical suspicion of DN or a clear history of diabetes generally do not receive renal biopsies, so the actual prevalence of DN is higher than reported in our study. We also found that the prevalence of DN was significantly greater during period 2 than period 1; this was probably due to the increasing prevalence of diabetes and the increasing use of renal biopsies to confirm DN. Because of the increasing prevalence of diabetes in China, we expected a higher prevalence of DN. We also found that the prevalence of HTARD significantly increased, but there was no obvious change in the prevalence of MHTARD. A previous epidemiological survey showed that the prevalence of hypertension in China increased from 18% in 2002 to 27.8% in 2013 [34], so it seems likely that renal damage caused by hypertension will continue to increase over time. We found that the prevalence of HBV-GN decreased from 10.9% (period 1) to 5.4% (period 2), similar to the results of Wang et al. [32].

The main clinical diagnoses of PGN were NS and CGN, consistent with previous reports in China [8], Brazil [24], and Europe [14, 35]. However, hematuria and proteinuria were the most common types among studies conducted in Hubei Province (China) [10], Korea [25], and Finland [36]. This difference may be related to slight differences in the indications for renal biopsy at different hospitals. In our study, MN (51.8%) and MCD (21.3%) were the main pathological types of NS, and IgAN was the most common pathological type of CGN. In agreement with our results, Zhou et al. found that MN (29.5%) and MCD (25.3%) were the most common types of NS [8], and a Japanese study reported that MN was the most common pathological type in NS (36.8%) [37].

## 5. Conclusions

Our study provides new information on the spectrum of kidney diseases and their changes in prevalence during the past decade in Northeast China. We also identified changes over time in patients in different age groups. The prevalence of MN increased, but the prevalence of IgAN decreased. There were also significant increases in the prevalence of DN, hypertension-associated renal damage, and systemic vasculitis-associated nephritis. In addition, different age groups had different changes in the prevalence of different pathological types of kidney diseases.

## Figures and Tables

**Table 1:** Pathological types of kidney diseases during different periods.

Pathological types	2008-12		2013-17		2008-17	
	N	(%)	N	(%)	N	(%)
PGN	1748	(72.1)	1845	(74.2)	3593	(73.2)
SGN	594	(24.5)	571	(23.0)	1165	(23.7)
TIN	73	(3.0)	66	(2.6)	139	(2.8)
HN	9	(0.4)	4	(0.2)	13	(0.3)
Total	2424	(100)	2486	(100)	4910	(100)

*Abbreviations*. *PGN*: primary glomerulonephritis; *SGN*: secondary glomerulonephritis; *TIN*: tubular-interstitial nephropathy; *HN*: hereditary nephropathy.

**Table 2:** Pathological types of SGN during different periods.

Pathological types	2008-12		2013-17		2008-17		*P* value
	N	(%)	N	(%)	N	(%)	
*Systemic Diseases*	348	(58.6)	266	(46.6)	614	(52.7)	<0.001
LN	209	(35.2)	187	(32.7)	396	(34.0)	0.380
HSPN	135	(22.7)	74	(13.0)	209	(17.9)	<0.001
SSARD	4	(0.7)	3	(0.5)	7	(0.6)	1.000
MCTARD	0	(0)	2	(0.4)	2	(0.2)	0.240
*Vascular diseases*	86	(14.5)	145	(25.4)	231	(19.8)	<0.001
HTARD	38	(6.4)	63	(11.0)	101	(8.7)	0.005
MHTARD	26	(4.4)	26	(4.5)	52	(4.4)	0.884
SVARD	14	(2.3)	54	(9.5)	68	(5.8)	<0.001
IN	7	(1.2)	2	(0.4)	9	(0.8)	0.201
HUS	1	(0.2)	0	(0)	1	(0.1)	1.000
*Metabolic diseases*	94	(15.8)	128	(22.4)	222	(19.1)	0.004
DN	53	(8.9)	83	(14.5)	136	(11.7)	0.003
AN	26	(4.4)	27	(4.7)	53	(4.5)	0.774
LCDD	2	(0.3)	8	(1.4)	10	(0.9)	0.099
C3GN	0	(0)	2	(0.4)	2	(0.2)	0.240
LPN	0	(0)	1	(0.2)	1	(0.1)	0.490
CUAN	1	(0.2)	0	(0)	1	(0.1)	1.000
OAG	8	(1.3)	4	(0.7)	12	(1.0)	0.423
ING	4	(0.7)	3	(0.5)	7	(0.6)	1.000
*Infectious diseases*	66	(11.1)	32	(5.6)	98	(8.4)	0.001
HBV-GN	65	(10.9)	31	(5.4)	96	(8.2)	0.001
HCV-GN	1	(0.2)	1	(0.2)	2	(0.2)	1.000
*Total*	594	(100)	571	(100)	1165	(100)	-

Abbreviations: *LN*, lupus nephritis; HSPN, Henoch-Schönlein purpura nephritis; *SSARD*, Sjogren's syndrome associated renal damage; *MCTARD,* Mixed connective tissue disease associated renal damage; *HT/MHTARD*, hypertension/malignant hypertension-associated renal damage; *SVARD*, systemic vasculitis-associated renal damage; *IN*, ischemic nephropathy; *HUS*, hemolysis aemolytic uraemic syndrome; *DN*, diabetic nephropathy; *AN*, amyloidosis nephropathy; *LCDD*, light chain deposition nephropathy; *C3GN*, C3 glomerulopathy; *LPN*, lipoprotein glomerulopathy; *CUAN*, chronic uric acid nephropathy; *OAG*, obesity-associated glomerulopathy; *ING*, idiopathic nodular glomerulosclerosis; *HBV-GN*, hepatitis B virus associated nephritis; *HCV-GN*, hepatitis C virus associated nephritis.

**Table 3:** Pathological types of kidney diseases in different age groups during different periods.

Age group	Pathological types	2008-12		2013-17		2008-17		*P *value
		N	(%)	N	(%)	N	(%)	
*≤24 yrs*	MN	54	(11.2)	38	(13.4)	92	(12.0)	0.356
	IgAN	117	(24.2)	71	(25.1)	188	(24.5)	0.788
	MCD	58	(12.0)	65	(23.0)	123	(16.1)	<0.001
	MsPGN	70	(14.5)	10	(3.5)	80	(10.4)	<0.001
	LN	50	(10.4)	35	(12.4)	85	(11.1)	0.391
	HSPN	51	(10.6)	20	(7.1)	71	(9.3)	0.108
	HT/MHTARD	4	(0.8)	5	(1.8)	9	(1.2)	0.414
	DN	1	(0.2)	0	(0)	1	(0.1)	1.000
	Others	78	(16.1)	39	(13.8)	117	(15.3)	0.379
*25-44 yrs*	MN	110	(11.0)	211	(25.1)	321	(17.4)	<0.001
	IgAN	375	(37.4)	241	(28.6)	616	(33.4)	<0.001
	MCD	61	(6.1)	77	(9.1)	138	(7.5)	0.013
	MsPGN	106	(10.6)	22	(2.6)	128	(6.9)	<0.001
	LN	103	(10.3)	97	(11.5)	200	(10.8)	0.393
	HSPN	46	(4.6)	17	(2.0)	63	(3.4)	0.002
	HT/MHTARD	34	(3.4)	39	(4.6)	73	(4.0)	0.174
	DN	9	(0.9)	23	(2.7)	32	(1.7)	0.003
	Others	158	(15.8)	115	(13.7)	273	(14.8)	0.204
*45-59 yrs*	MN	157	(23.5)	425	(50.0)	582	(38.4)	<0.001
	IgAN	138	(20.7)	82	(9.6)	220	(14.5)	<0.001
	MCD	41	(6.1)	61	(7.2)	102	(6.7)	0.427
	MsPGN	57	(8.5)	14	(1.6)	71	(4.7)	<0.001
	LN	52	(7.8)	37	(4.4)	89	(5.9)	0.005
	HSPN	27	(4.0)	18	(2.1)	45	(3.0)	0.028
	HT/MHTARD	19	(2.8)	29	(3.4)	48	(3.2)	0.534
	DN	30	(4.5)	38	(4.5)	68	(4.5)	0.980
	Others	146	(21.9)	146	(17.2)	292	(19.2)	0.021
*≥60 yrs*	MN	94	(34.6)	248	(48.5)	342	(43.7)	<0.001
	IgAN	23	(8.5)	26	(5.1)	49	(6.3)	0.064
	MCD	21	(7.7)	50	(9.8)	71	(9.1)	0.338
	MsPGN	25	(9.2)	8	(1.6)	33	(4.2)	<0.001
	LN	4	(1.5)	18	(3.5)	22	(2.8)	0.154
	HSPN	11	(4.0)	19	(3.7)	30	(3.8)	0.821
	HT/MHTARD	7	(2.6)	16	(3.1)	23	(2.9)	0.660
	DN	13	(4.8)	22	(4.3)	35	(4.5)	0.760
	Others	74	(27.2)	104	(20.4)	178	(22.7)	0.029
*All ages*	MN	415	(17.1)	922	(37.1)	1337	(27.2)	<0.001
	IgAN	653	(26.9)	420	(16.9)	1073	(21.9)	<0.001
	MCD	181	(7.5)	253	(10.2)	434	(8.8)	0.001
	MsPGN	258	(10.6)	54	(2.2)	312	(6.4)	<0.001
	LN	209	(8.6)	187	(7.5)	396	(8.1)	0.157
	HSPN	135	(5.6)	136	(5.5)	271	(5.5)	0.880
	HT/MHTARD	64	(2.6)	89	(3.6)	153	(3.1)	0.058
	DN	53	(2.2)	83	(3.3)	136	(2.8)	0.014
	Others	456	(18.8)	342	(13.8)	798	(16.3)	<0.001

**Table 4:** Clinical manifestations and pathological types of PGN.

Pathological types	AGN		CGN		NS		RPGN		AUA		Total
	N	(%)	N	(%)	N	(%)	N	(%)	N	(%)	
IgAN	1	(3.8)	799	(55.6)	196	(9.9)	0	(0)	77	(63.1)	1073
MsPGN	0	(0)	90	(6.3)	214	(10.8)	0	(0)	8	(6.6)	312
MN	0	(0)	305	(21.2)	1026	(51.8)	0	(0)	6	(4.9)	1337
FSGS	0	(0)	46	(3.2)	64	(3.2)	0	(0)	0	(0)	110
MCD	0	(0)	0	(0)	422	(21.3)	0	(0)	0	(0)	123
GML	0	(0)	91	(6.3)	1	(0.1)	0	(0)	31	(25.4)	422
CreGN	0	(0)	1	(0.1)	0	(0)	29	(100)	0	(0)	30
MPGN	2	(7.7)	16	(1.1)	37	(1.9)	0	(0)	0	(0)	55
EnPGN	23	(88.5)	0	(0)	0	(0)	0	(0)	0	(0)	23
Others	0	(0)	89	(6.2)	19	(1.0)	0	(0)	0	(0)	108
*Total*	*26*	*(100)*	*1437*	*(100)*	*1979*	*(100)*	*29*	*(100)*	*122*	*(100)*	*3593*

## Data Availability

The data used to support the findings of this study are available from the corresponding author upon request.
